# Preparation and optimization of surface stabilized cryptotanshinone nanocrystals with enhanced bioavailability

**DOI:** 10.3389/fphar.2023.1122071

**Published:** 2023-02-03

**Authors:** Wenzheng Zhao, Bohao Ruan, Xiaoyi Sun, Zhenwei Yu

**Affiliations:** ^1^ Sir Run Run Shaw Hospital, College of Medicine, Zhejiang University, Hangzhou, China; ^2^ Department of Pharmacy, Hangzhou City University, Hangzhou, China; ^3^ Institute of Pharmaceutics, College of Pharmaceutical Sciences, Zhejiang University, Hangzhou, China

**Keywords:** cryptotanshinone, nanocrystals, pharmacokinetics, poloxamer 407, oral administration

## Abstract

Cryptotanshinone (CTS) is a plant product extracted from *Salvia miltiorrhiza Bunge* with various pharmacological significances. In addition to its activities against coronary heart disease, hyperlipidemia, stroke, hepatitis and chronic renal failure, it demonstrates antimetastatic effects. However, its clinical use is limited due to its poor aqueous solubility and oral bioavailability. Herein, CTS nanocrystals were prepared with the precipitation method followed by high-pressure homogenization using Poloxamer 407 as the stabilizer. A stable product was further obtained by lyophilization. The particle size of the CTS nanocrystals was 315.67 ± 11.02 nm, and the zeta potential was near 0 mV. The crystallinity was confirmed by DSC and PXRD. The saturation solubility was substantially increased from 0.97 ± 0.12 μg/ml to 62.29 ± 1.91 μg/ml, and the dissolution rate was also significantly accelerated. A pharmacokinetic study in rats revealed an improvement in oral bioavailability (2.87-fold) with CTS nanocrystals compared to the raw drug. In conclusion, the results of this study suggest a feasible formulation for the oral delivery of CTS.

## 1 Introduction

**FIGURE 1:**
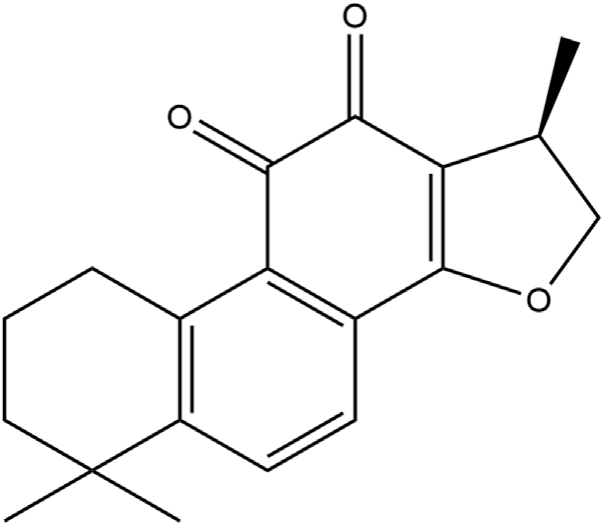
The chemical structure of cryptotanshinone (1,2,6,7,8,9-hexahydro-1,6,6-trimethyl-(R)-phenanthro (1,2-b)furan-10,11-dione).

Cryptotanshinone (CTS, Figure 1), also named cryptotanshinone or tanshinone C, is an important diterpene quinone isolated from the roots and rhizomes of the plant *Salvia miltiorrhiza Bunge* (Li H. et al., 2021). CTS has been reported to show a variety of pharmacological activities, such as neuroprotective, antineoplastic, anti-inflammatory, antioxidant, and radioprotective effects, to treat multiple diseases and disorders, including acute ischemic stroke, atherosclerosis, Alzheimer’s disease, diabetes, obesity, chronic hepatitis and cancer (Wu et al., 2020; Ashrafizadeh et al., 2021).

Despite these comprehensive advantages, no CTS preparation is currently available on the market because of its low solubility in water. Although the logP of CTS is 4.13 which indicates its good permeability, its 9.76 μg/ml solubility in water (Zhang et al., 2006) hinders the oral bioavailability of this BCS II compound (Bhalani et al., 2022), evidenced by 2.05% of the absolute oral bioavailability in rats (Zhang et al., 2006). Oral administration of 20 mg/kg CTS in pigs resulted in non-detectable drug in serum, while only 0.043 μg/ml CTS was detected 1 h after administration when the dose increased to 40 mg/kg (Xue et al., 1999). Researchers to date have tried to use solid lipid nanoparticles (Hu et al., 2010), lipid-polymer hybrid nanoparticles (Liu et al., 2020), solid dispersions (Zhai et al., 2017), cyclodextrin inclusions (Li et al., 2003), solid self-microemulsifying drug delivery systems (Bi et al., 2016) and other technologies to increase the solubility of CTS for oral administration (Cai et al., 2016). Nevertheless, the technologies above have strict requirements for the physical and chemical characteristics of drug molecules, resulting in low drug loading, low entrapment efficiency and premature drug release. The composition of the above preparations is complex, suitable carriers are needed, and toxic excipients such as Tween 80™ and sodium dehydrocholate (Hu et al., 2010) may be used. Moreover, the preparation process of these technologies is complicated and difficult to industrialization, which hinders their clinical translation.

In the past 3 decades, drug nanocrystals, as an emerging technology that could enhance the dissolution and bioavailability of compounds belonging to BCS II, have sprung up and attracted extensive attention from pharmaceutical researchers (Verma et al., 2009). Drug nanocrystals are insoluble drug particles that form inhomogeneous water dispersions with particle sizes of 1–1000 nm (Li J. et al., 2021). According to the Noyes–Whitney equation, a decrease in particle size will lead to an increase in the effective surface area in the diffusion layer, thus increasing the dissolution rate of drugs (Noyes and Whitney, 1897). Morover, drug nanocrystals can improve adhesiveness to the gut wall and diffusion to intestinal water layers (Imono et al., 2020). Different from other formulation strategies, drug nanocrystals do not require a carrier and are composed of almost 100% drug particles and a minimum amount of suitable surfactants and/or polymers as stabilizers (Hou et al., 2017). Meanwhile, the high drug loading of nanocrystals increases patient compliance (Li J. et al., 2021), eliminates food effects and improves efficacy and safety for oral administration (Wang et al., 2022). The production methods, including solvent-antisolvent precipitation, high-pressure homogenization and media milling, are universal and simple for large-scale production with excellent reproducibility (Chen et al., 2011). Although this technology was developed in the 1970s, more than 20 nanocrystal-based drug products have been approved for analgesia, schizophrenic, antifungal, antitumor and anti-inflammatory treatments, and several clinical trials are currently undergoing at different stages (Fan et al., 2018). The research revealed that the submissions received by the FDA for nanocrystal drug products comprise approximately 30% of all the applications for drug products containing nanomaterials (Chen et al., 2017), which fully confirms the broad prospects of nanocrystal preparations.

Herein, freeze-dried CTS nanocrystals were successfully developed by a combination approach with precipitation followed by high-pressure homogenization to improve the oral bioavailability of CTS. The physicochemical characteristics, including particle size, crystal morphology, crystalline state, thermodynamic properties, solubility, drug release profiles and stability, were determined. The pharmacokinetics of CTS nanocrystals were evaluated in rats.

## 2 Materials and methods

### 2.1 Materials

Cryptotanshinone (≥98%, batch No. 20200617) was purchased from Shanxi Angsheng Biopharmaceutical Co., Ltd. (Shanxi, China). Poloxamer 407 (batch No. BCCB1080) was purchased from BASF (Ludwigshafen, Germany). Fenofibrate (batch No. O0501A) was bought from Meilunbio (Dalian, China). HPLC-grade methanol was obtained from Sigma‒Aldrich (St. Louis, MO, United States). All other reagents were of analytical grade.

Male Sprague‒Dawley rats (180–220 g) were provided by Shanghai Slac Laboratory Animal Co. Ltd. (Shanghai, China). The animal qualification number was SCXK (Hu) 2017–0005. All animal experiments were performed in accordance with the guidelines for the welfare and ethics of experimental animals of Zhejiang University with the approval of the Animal Experimental Ethics Committee of Zhejiang University (ZJU20210258).

### 2.2 Preparation of cryptotanshinone nanocrystals

CTS nanosuspensions were produced by precipitation combined with high-pressure homogenization. Briefly, 300 mg CTS in acetone was added dropwise into 100 ml 1% poloxamer 407 aqueous solution under 800 rpm/min magnetic stirring at room temperature. Then, the crude dispersion formed with further stirring for 4 h and passed through a high-pressure homogenizer (ATS-AH2010, Brampton, ON, CAN) for 5 cycles at 300 bar and finally 8 cycles at 1,000 bar to obtain uniform small particles. Mannitol was used as the cryoprotectant. The freeze-dried nanocrystals were obtained by lyophilization process (CHRIST Alpha 2-4 LSC, Osterode, GER): prefrozen at -40°C for 8 h, then shelf temperature was raised to -20°C for 8 h, -20°C–0°C for 12 h, secondary drying was set at 25°C for 5 h.

### 2.3 Characterization of cryptotanshinone nanocrystals

#### 2.3.1 Particle size and zeta potential

The particle size and zeta potential of the nanocrystals were determined by a Malvern Instrument (Zetasizer Nano ZS90, Malvern, United Kingdom). The nanosuspension was diluted 20 times with distilled water before measurement.

#### 2.3.2 Transmission electron microscopy

TEM (JEM-1230, JEOL, JP) was used to observe the morphology of nanocrystals without staining.

#### 2.3.3 *In vitro* release

*In vitro* release of CTS nanocrystals was performed using dialysis against 1% sodium dodecyl sulfate (SDS) in 20 ml pH progressive dissolution media (HCl buffer pH 1.2 for 2 h followed by phosphate buffered saline (PBS, pH 6.8). Lyophilized nanocrystals (1 mg CTS) were redispersed in buffers and placed in a dialysis bag (MWCO 14 kDa). Dialysate was collected and analyzed by HPLC (Alliance 2690; Waters, Milford, MA, United States). The detection conditions were referred to a published validated method with minor modifications as follows: Diamonsil C_18_ column (250 × 4.6 mm, 5 µm); flow rate 1 ml/min, wavelength 269 nm. The mobile phase was methanol:water (75:25), column temperature 30°C. Linearity was good in the range of 0.75–50 μg/ml (y = 36,612x+13,691, R^2^ = 0.9945) (Chu et al., 2014).

#### 2.3.4 Powder X-ray diffraction and differential scanning calorimetry

The crystalline state of the powders was determined by a diffractometer (D8 Advance, Bruker, GER). The PXRD patterns of the samples were obtained in the 2θ range of 3°–40° with a scanning rate of 6°/min. A differential scanning calorimeter (Q100, TA, United States) was used to investigate the thermal behavior of pure CTS, CTS nanocrystals, blank excipient, and a physical mixture. Samples were separated, sealed in aluminum pans and heated between 25°C and 275°C at a rate of 10°C/min.

#### 2.3.5 Solubility

The aqueous solubility of CTS and CTS nanocrystals was determined in HCl buffer at pH 1.2 (simulated gastric pH), phosphate buffer at pH 6.8 (simulated intestinal pH), and distilled water at 37°C.

#### 2.3.6 Stability study

The physical and chemical stabilities of lyophilized nanocrystals stored at 4°C and 25°C for 6 months were evaluated to identify their storage recommendation. All the samples were monitored for particle size, zeta potential, polydispersity index, and CTS content after redispersion in water at intervals of 1, 3, and 6 months. The CTS assay was determined by HPLC.

### 2.4 Pharmacokinetics study in rats

Ten rats were separated into a raw CTS-treated group and a CTS nanocrystal-treated group. Suspensions were prepared by dispersing bulk CTS and redispersing lyophilized CTS nanocrystals in saline (0.9% NaCl). Rats were randomly orally administered a single dose of 20 mg/kg. At different time points of 0.25, 0.5, 0.75, 1, 2, 4, 6, 8, and 12 h, blood samples (0.5 ml) were taken from the tail vein into heparin-containing tubes. The samples were centrifuged to separate plasma.

The plasma concentration of CTS was determined by LC‒MS/MS. Briefly, an aliquot of 0.1 ml plasma was mixed with 10 µL fenofibrate methanol solution (internal standard) and 1 ml acetonitrile. The samples were mixed and centrifuged at 12,000 rpm for 10 min. The supernatant was collected and assayed by a published LC‒MS/MS method (Song et al., 2005).

The instrumental system for LC‒MS/MS consisted of an Agilent 1290 LC system and an Agilent 6400 triple quadrupole tandem mass spectrometer (Agilent Technologies, Waldbronn, Germany). A reversed-phase HPLC column (Waters-C_18_ column; 4.6 mm × 250 mm, 5 µm) was used for chromatographic separation. The mobile phase was methanol:water (85:15), and the flow rate was set at 1 ml/min. The column eluent was monitored by a mass spectrometer equipped with atmospheric pressure chemical ionization (APCI) in positive ion mode. The instrumental conditions were as follows: corona current, 6 μA; vaporizer temperature, 500 °C; nebulizer current, 6.0 µA; and collision energy, 25 eV. The precursor ([M + H]^+^)-to-product ion transitions used for quantification were *m/z* 297→251 for CTS and *m/z* 361.1→233 for the IS.

The concentration-time profile data were fitted, and the pharmacokinetic parameters were calculated by statistical moment. Peak concentration (C_max_) and time of peak concentration (T_max_) were obtained directly from the individual plasma concentration-time curves. K_e_ is the elimination rate constant calculated from the terminal slope of the plasma concentration-time curves. AUMC and AUC from time zero to infinity were calculated by the trapezoidal method. The mean residence time (MRT) was obtained by AUMC_0-∞_/AUC_0-∞_.

## 3 Results

### 3.1 Optimization of CTS nanocrystal formulation

**FIGURE 2:**
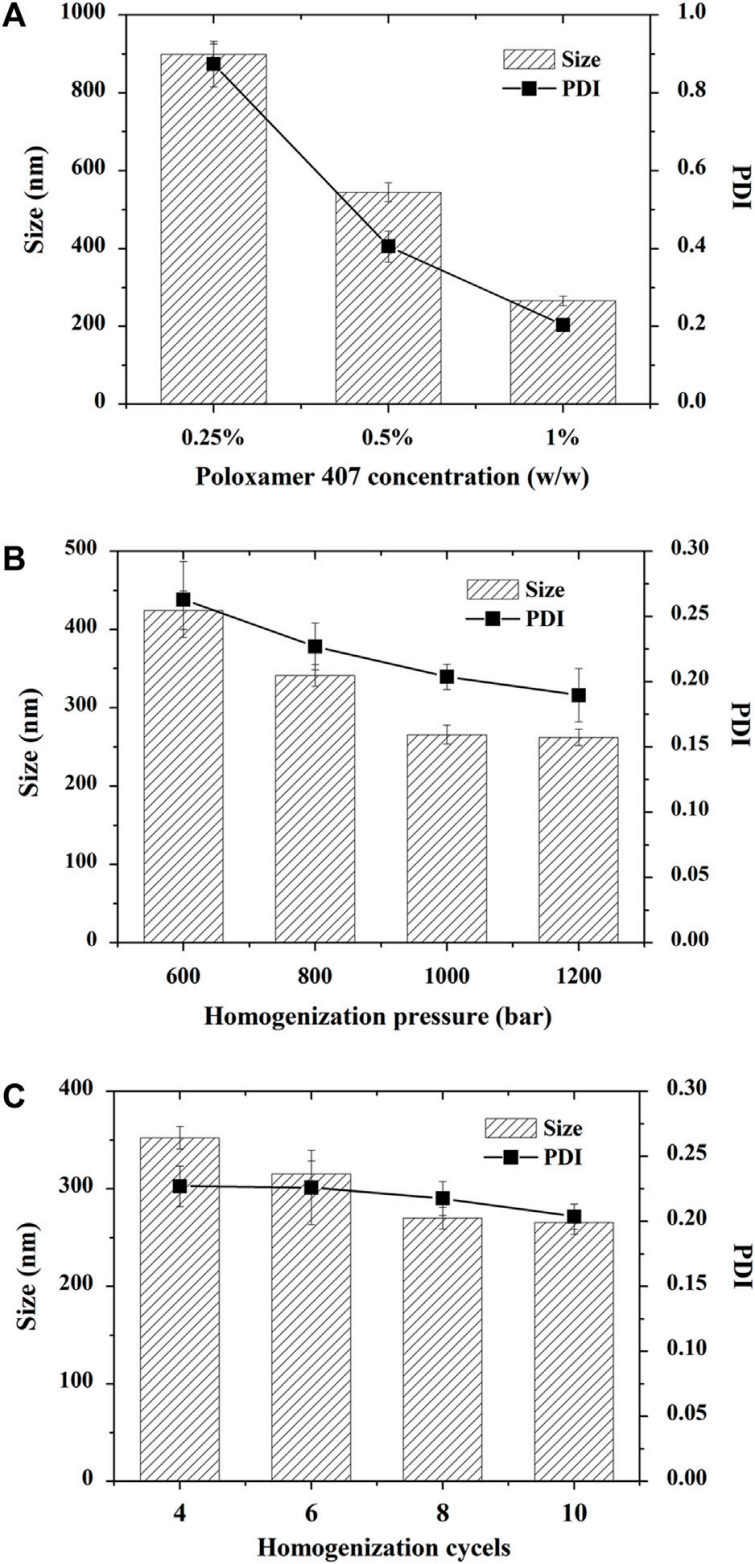
Optimization of cryptotanshinone (CTS) nanocrystals. All the particles were prepared by precipitation combined with high-pressure homogenization. **(A)** Effect of poloxamer 407 on the particle size and PDI of CTS nanocrystals; **(B)** Effect of homogenization pressure on the particle size and PDI of CTS nanocrystals; **(C)** Effect of homogenization cycles on the particle size and PDI of CTS nanocrystals. The data represent the mean ± SD, *n* = 3.

In the preliminary study, HPMC, PVP, poloxamer 188, and poloxamer 407 were utilized as stabilizers to produce CTS nanosuspensions. The crystals go beyond micrometers in the presence of HPMC, PVP and poloxamer 188 (data not shown). The nanosuspension prepared by Poloxamer 407 hardly produced sedimentation, and the particle size was less than 1 µm. Subsequently, the concentration of Poloxamer 407 was optimized to achieve a fine particle size (Figure 2A). The particle size ranged from 265.67 ± 12.01 nm to 899.33 ± 26.31 nm upon altering the stabilizer concentration from 0.25% to 1%. The polydispersity index (PDI) increased along with the particle size ranging from 0.20 ± 0.01 to 0.87 ± 0.06.

The homogenization pressure and number of cycles are crucial to reducing the particle size in the top-down technique. We observed that increasing the homogenization pressure led to a significant decrease in nanocrystal size from 424.33 ± 24.54 nm to 265.67 ± 12.01 nm, followed by a gradual reduction when the pressure exceeded 1,000 bars (Figure 2B). Similarly, when we fixed the homogenization pressure at 1,000 bars, increasing the homogenization cycles from 4 to 10 cycles reduced the CTS particle size from 352.33 ± 11.50 nm to 265.67 ± 12.01 nm (Figure 2C). When the homogenization cycle exceeded 8 cycles, the particle size hardly changed. Finally, the optimized formulation was prepared by homogenizing CTS nanosuspensions in 1% poloxamer 407 for 8 cycles at 1,000 bar. The zeta potential of CTS nanosuspensions was near 0 (0.07 ± 0.17 mV).

### 3.2 Morphology

**FIGURE 3:**
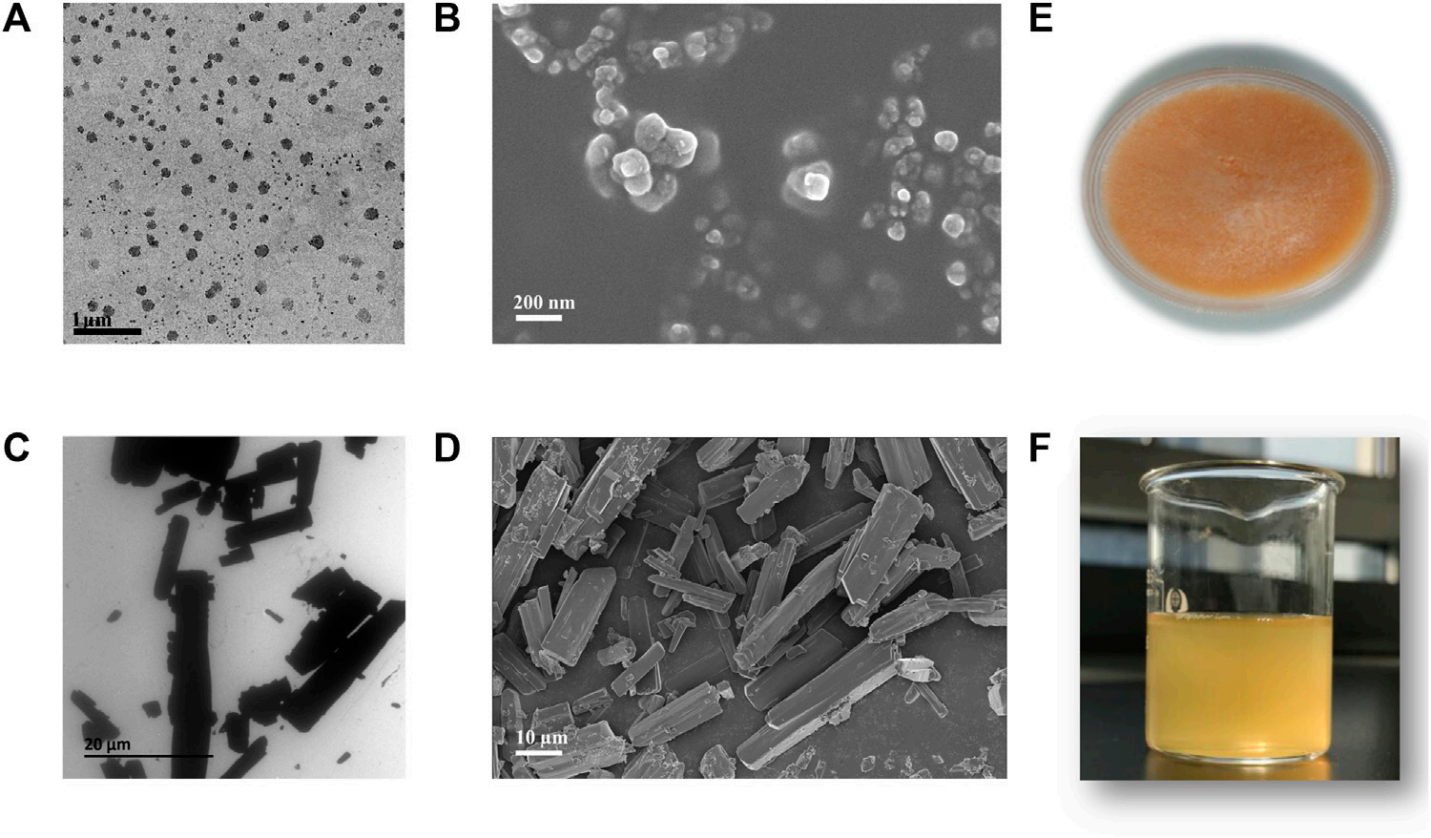
Morphology and appearance of bulk cryptotanshinone (CTS) and CTS nanocrystals. **(A)** Transmission electron micrographs and **(B)** scanning electron micrographs of CTS nanocrystals; **(C)** transmission electron micrographs and **(D)** scanning electron micrographs of bulk CTS; **(E)** freeze-dried product of CTS nanocrystals; **(F)** rehydrated CTS nanosuspension.

Microscopy images revealed that CTS nanocrystals were homogeneous spherical or nearly spherical with a size of approximately 200 nm. They were well dispersed without agglomerations (Figures 3A, B). In contrast, the bulk CTS were rod-shaped microparticles at the micrometer scale (Figures 3C, D). After reconstitution of lyophilized CTS nanocrystals with water (Figure 3E), the CTS nanodispersion was a uniform yellow suspension (Figure 3F).

### 3.3 PXRD analysis

**FIGURE 4:**
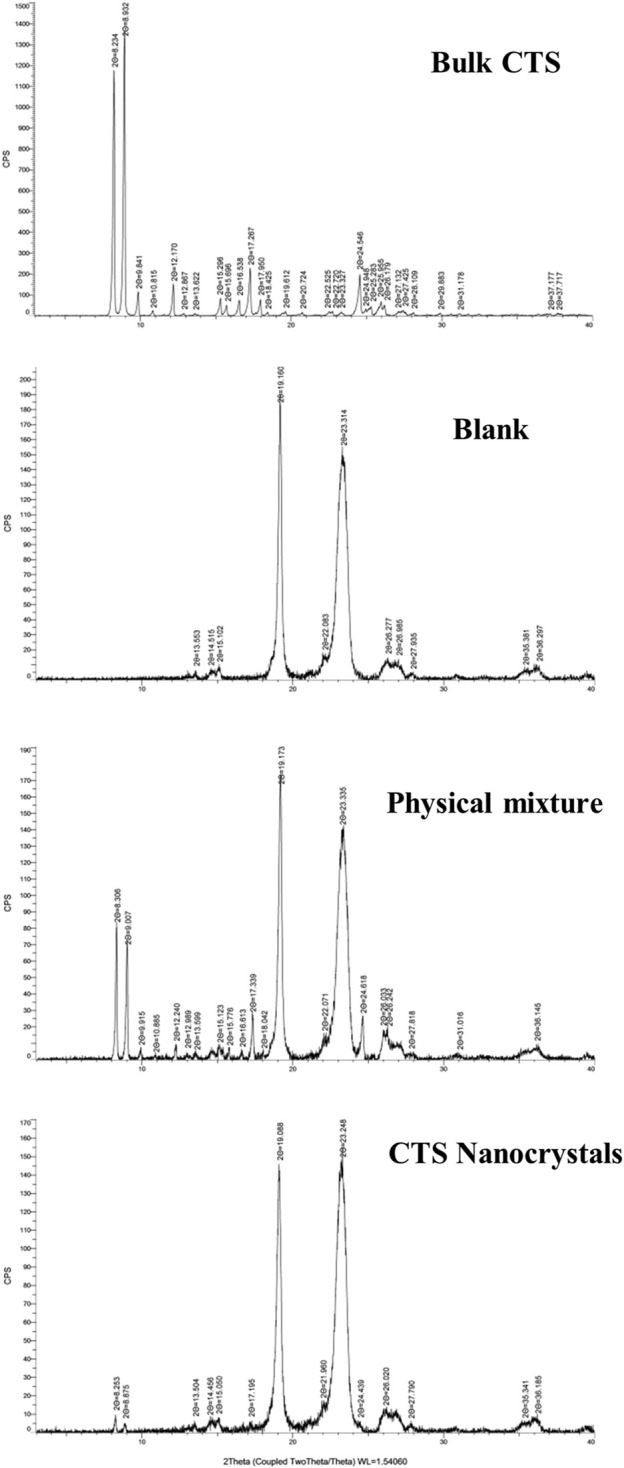
PXRD spectra of bulk cryptotanshinone (CTS), the excipients (blank), physical mixture and CTS nanocrystals.

PXRD spectra of CTS, CTS nanocrystals, the physical mixture, and the blank excipient (Blank) are shown in Figure 4. Intense and sharp diffraction peaks at 2θ values between 8° and 31° indicated the crystalline property of pure CTS. Characteristic peaks with lower intensity in the same range were observed by the optimized CTS formulation, especially at 2θ values of 9.2° and 8.9°. The results demonstrated that the crystalline state of CTS nanocrystals was preserved and that there were no transformations during high-pressure homogenization. However, reduced crystallinity occurred in terms of the broadening peak and reduction in peak intensity.

### 3.4 Thermal analysis

**FIGURE 5:**
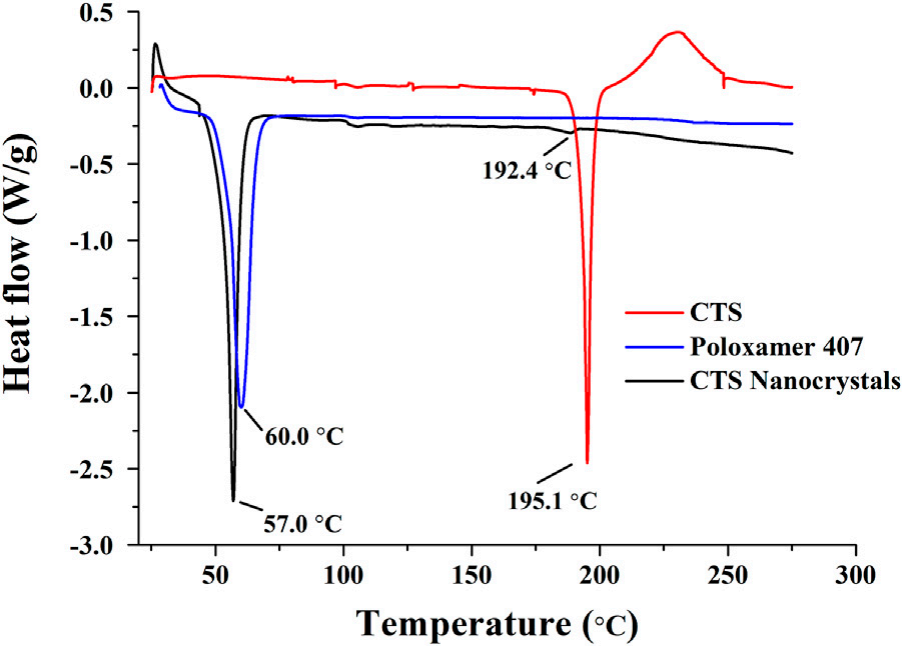
DSC thermograms of cryptotanshinone (CTS), poloxamer 407 and CTS nanocrystals.

The DSC thermograms are shown in Figure 5. CTS and stabilizer poloxamer 407 displayed characteristic sharp endothermic peaks at 195.1°C and 60.0°C, respectively. Lyophilized CTS nanocrystals exhibited characteristic peaks slightly shifted to 192.4°C and 57.0°C, which corroborated the presence of CTS and poloxamer 407 in the final product. However, the density of the drug-based peak dramatically decreased, showing the partial amorphization of CTS.

### 3.5 Solubility and dissolution studies

**FIGURE 6:**
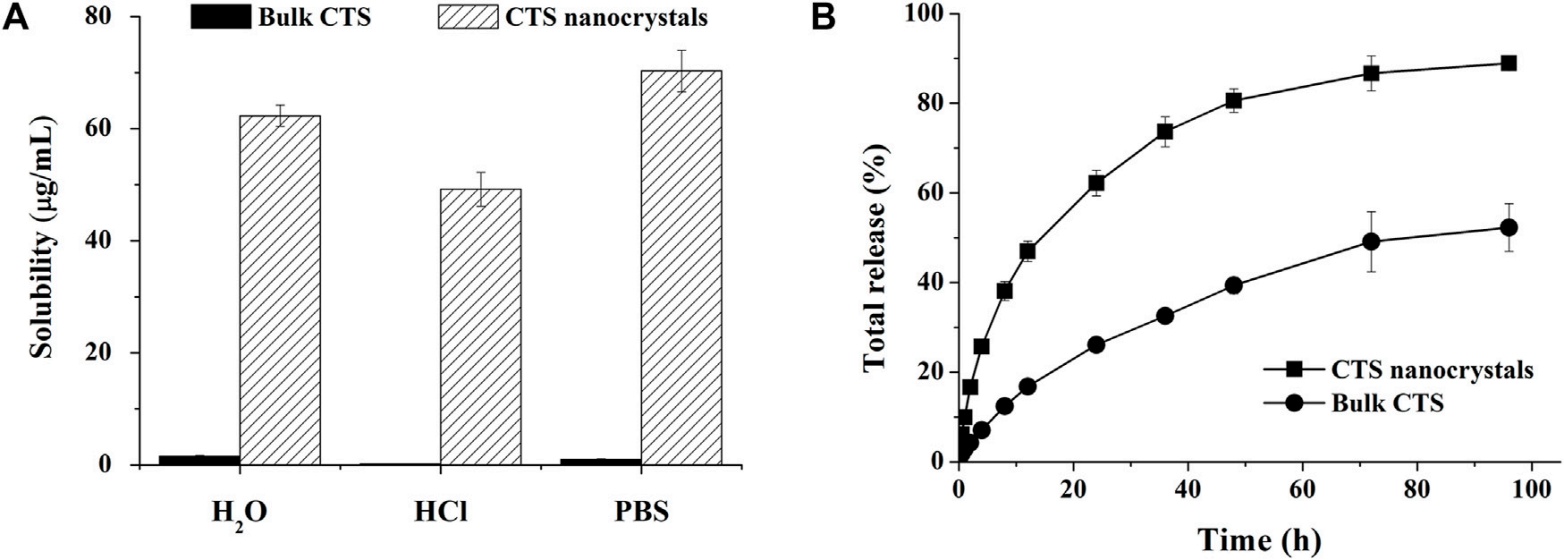
Improved solubility and dissolution rate by cryptotanshinone (CTS) nanocrystals. **(A)** The solubility of bulk CTS and CTS nanostructures in different media; **(B)** *In vitro* release profile of bulk CTS and CTS nanostructures in pH-progressive media. The data represent the mean ± SD, *n* = 3.

The solubilities of bulk CTS and lyophilized nanocrystals in aqueous solutions with different pH values are shown in Figure 6A. The saturation solubility of CTS was extremely improved in water (∼60-fold), HCl buffer (pH 1.2, ∼200-fold) and phosphate buffer (pH 6.8, ∼40-fold). The solubility of raw CTS was 4.15 ± 0.03 μg/ml, 4.11 ± 0.02 μg/ml, and 5.22 ± 0.01 μg/ml in 1% Poloxamer 407 (aqueous solution), 1% Poloxamer 407 (HCl buffer), and 1% Poloxamer 407 (PBS buffer), respectively. Compared with the solubility in water, HCl and PBS, the solubility can be slightly increased to 2.6, 16.2, 5.4 times by 1% Poloxamer 407. Poloxamer 407 contributed to the enhanced solubility of CTS, but nanocrystals contributed more.

An *in vitro* release study was performed in pH progressive media (Figure 6B). The nanosized CTS displayed an accelerated and intense release compared with bulk CTS. Nanocrystals released 89% of the total drug within 96 h, while pure CTS only achieved 52% drug dissolution. Zero-order, first-order, Higuchi, Ritger-Peppas, and Hixcon-Crowell models were performed to fit the release profiles of CTS nanocrystals and bulk CTS (Table 1). Higuchi kinetics showed the best goodness of fit (R^2^ = 0.9906 for nanocrystals and R^2^ = 0.9932 for bulk CTS), indicating the diffusion controlled principal. The diffusion rate constant (*K*_*H*_) was calculated to be 12.393 and 5.819 for nanocrystals and bulk CTS, respectively which showed the increased release rate (2.13-fold) by nanocrystals.

**TABLE 1:** Model fitting results for the release profiles of CTS nanocrystals and bulk CTS.

Models	CTS nanocrystals		Bulk CTS	
	Fitting equation	R^2^	Fitting equation	R^2^
Zero-order	$M_{t}/M_{\infty }=0.0096t+0.1898$	0.7886	$M_{t}/M_{\infty }=0.0058t+0.0524$	0.9285
First-order	$\ln\left(1-M_{t}/M_{\infty }\right)=-0.0245t-0.1922$	0.9426	$\ln\left(1-M_{t}/M_{\infty }\right)=-0.0082t-0.0466$	0.9662
Higuchi	$M_{t}/M_{\infty }=12.393t^{1/2}-0.544$	0.9906	$M_{t}/M_{\infty }=5.8194t^{1/2}-2.5966$	0.9932
Ritger-Peppas	$\ln\left(M_{t}/M_{\infty }\right)=0.5707\ln t+2.2217$	0.9601	$\ln\left(M_{t}/M_{\infty }\right)=0.6644\ln t+1.0697$	0.9971
Hixcon-Crowell	${\left(100-M_{t}/M_{\infty }\right)}^{1/3}=-0.0268t+4.3325$	0.8974	${\left(100-M_{t}/M_{\infty }\right)}^{1/3}=-0.0113t+4.5654$	0.9550

Note: $M_{t}/M_{\infty }$ is the percent of drug released at time *t*.

### 3.6 Stability study

The physical and chemical stabilities of the lyophilized products were evaluated for 6 months at 4°C and 25°C, respectively (Table 2). The original particle size was 315.67 ± 11.02 nm. There were no remarkable changes in particle size, PDI and drug content up to 6 months of storage at 4°C, indicating that no partially amorphous to crystalline conversion occurred. However, a considerable increase in particle size was observed following 3–6 months of storage at room temperature. These results revealed that CTS was chemically stable at both 4°C and 25°C. However, they should be stored at 4°C for crystal growth.

**TABLE 2:** Stability test of lyophilized cryptotanshinone (CTS) nanocrystals at 4°C and 25°C.

Temperature	Time (months)	Particle size (nm)	PDI	CTS Content (%)
**__**	0	315.67 ± 11.02	0.20 ± 0.02	100
4°C	1	321.67 ± 10.41	0.21 ± 0.02	101.14 ± 7.59
	3	310.33 ± 12.50	0.21 ± 0.02	103.02 ± 4.83
	6	325.33 ± 15.57	0.19 ± 0.01	98.59 ± 5.37
25°C	1	312.00 ± 14.53	0.21 ± 0.02	101.57 ± 3.97
	3	330.67 ± 16.01	0.22 ± 0.02	96.75 ± 3.97
	6	341.33 ± 13.05	0.24 ± 0.03	102.81 ± 6.86

The data represent the mean ± SD, n = 3.

### 3.7 Pharmacokinetics

**FIGURE 7:**
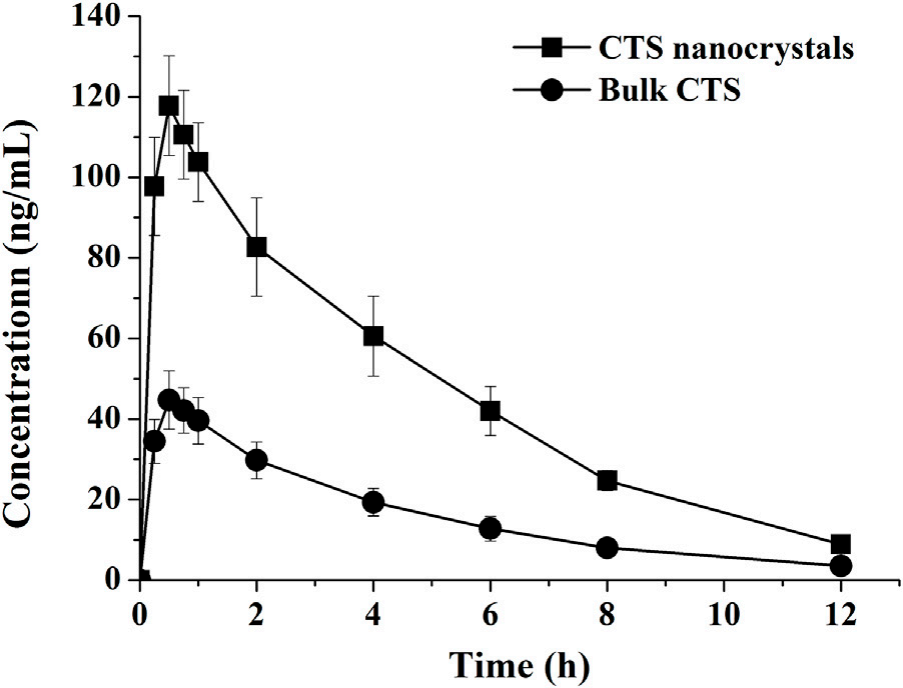
Plasma concentration-time curves of bulk cryptotanshinone (CTS) and CTS nanocrystals. Values represent mean ± SD, *n* = 5.

The pharmacokinetic profiles of the CTS suspension and nanocrystals after a single oral dose in healthy rats are plotted in Figure 7, and the pharmacokinetic parameters were calculated using a non-compartmental model (Table 3). Compared with the raw CTS suspension (45.5 ± 6.6 ng/ml), CTS nanocrystals resulted in a higher C_max_ (118.7 ± 11.2 ng/ml, *p < 0.01*). The ACU_0-∞_ of CTS nanocrystals (607.0 ± 62.7 ng h/ml) was significantly higher than that in the CTS suspension group (211.58 ± 30.58 ng h/ml, *p < 0.01*). Absorption was slightly accelerated in terms of the shortening of T_max_ from 0.5 ± 0.2 h to 0.4 ± 0.1 h by the nanoformulation. The relative bioavailability was 286.87%. These results indicated that CTS nanocrystals had a higher absorption rate and improved bioavailability than raw CTS.

**TABLE 3:** Pharmacokinetic parameters of cryptotanshinone (CTS) suspension and CTS nanocrystals after oral administration to rats.

Parameters	CTS Suspension	CTS Nanocrystals
Ke (h^−1^)	0.22 ± 0.02	0.23 ± 0.02
T_1/2_ (h)	3.24 ± 0.25	3.08 ± 0.21
T_max_ (h)	0.45 ± 0.21	0.40 ± 0.14
C_max_ (ng/ml)	45.52 ± 6.56	118.70 ± 11.18**
MRT (h)	4.67 ± 0.32	4.62 ± 0.22
AUC_0-∞_ (ng·h/mL)	211.58 ± 30.58	606.95 ± 62.65**
Relative Bioavailability (%)	-	286.87

Data is presented as mean ± SD. ** indicated *p* <0.01 as compared with CTS suspension group.

## 4 Discussion

It is the first attempt to develop lyophilized CTS nanocrystals and its enhanced oral bioavailability was also well evaluated. It is proved that optimized CTS nanocrystals had remarkable enhancement in solubility, dissolution rate and bioavailability. Our result indicated that nanocrystals are a suitable delivery approach for CTS oral administration.

The strategies for nanocrystal fabrication are divided into top-down, bottom-up methods, and combination techniques. In the case of top-down methods, milling and homogenization are currently utilized to reduce microsized drug crystal size with the aid of physical forces. Bottom-up methods such as solvent-antisolvent precipitation, supercritical fluids, and solvent evaporation are employed to prepare homogenous and small particles. The combination of both approaches, usually involving bottom-up and top-down processes in sequence, favors the production of more homogenous and smaller nanocrystals (Fontana et al., 2018). Herein, we first dissolved CTS in water-miscible acetone and then precipitated it by the addition of a stabilizer-containing antisolvent under rapid mixing to obtain fine crystalline CTS. Solvent-antisolvent precipitation refers to the nucleation and precipitation of API under supersaturation conditions. A more concentrated drug solution leads to a higher number of nuclei with smaller particles generated (Lonare and Patel, 2013). Therefore, the drug concentration in acetone was set as the saturated concentration. Other variables that impact drug crystals formed by precipitation are stabilizers (Matteucci et al., 2006). A suitable stabilizer is able to provide favorable surface covering and steric stabilization on the newly formed surface area of the nanosuspension (Sattar et al., 2017; Ullah et al., 2018). We found that poloxamer 407 was the best among these commonly used stabilizers. Poloxamers are non-ionic polymers consisting of hydrophilic ethylene oxide and relatively hydrophobic propylene oxide blocks with good biocompatibility and non-toxicity. In addition to the particle‒particle repulsion provided by hydrophilic chains from polymers HPMC and PVP, amphiphilic poloxamers can additionally offer adequate physical adsorption on the precipitated hydrophobic drug surface by their hydrophobic domains. The optimal stabilizer is mainly dependent on the similarity of hydrophobicity between the drug and stabilizer (Shegokar and Müller, 2010). Compared with poloxamer 188 (20%), poloxamer 407 has a higher weight ratio of polyoxypropylene chain segments (30%). It can diffuse to and adsorb readily on the CTS surface to reduce the high surface free energy, promote the nucleation rate, and impede crystal growth by coagulation and condensation (Matteucci et al., 2006). Subsequently, the concentration of poloxamer 407, homogenization pressure and number of cycles were optimized to achieve a desirable particle size. Smaller particles would be obtained with a higher concentration of stabilizer owing to the insufficiency of the poloxamer to arrest crystal growth. However, a further increase in the stabilizer resulted in larger crystals because the viscosity of the aqueous solution increased (Sharma and Mehta, 2019). We obtained CTS nanocrystals with an average particle size of 315.67 ± 11.02 nm and a polydispersity index of 0.20 ± 0.02 when 1% poloxamer 407 was used. A small particle size (∼300 nm) and narrow size distribution (PDI <0.25) are usually considered acceptable for nanocrystal production (Patravale et al., 2004). Homogenization disrupts drug particles to nanoscale dimensions with shear and impact forces. Obviously, high energy inputs, such as higher homogenization pressure and an increase in homogenization cycles, would reduce the average particle size (Figures 2B, C). However, further increases in the homogenization pressure and number of cycles did not decrease the particle size impeded by enhanced recoalescence.

Lyophilization is a suitable solidification technique to transform nanosuspensions into a stable and easily redispersible formulation. As the conventional cryoprotectant, CTS nanocrystals lyophilized with mannitol exhibited quick reconstitution in water (<30 s) without agglomerates (Table 2). Neither Ostwald ripening nor aggregation was observed up to 6 months of storage at 4°C, as evidenced by the unchanged particle size and PDI value. The characteristic peaks of CTS nanocrystals in the diffractogram showed that the preparation process, including precipitation, homogenization, and lyophilization, did not change the drug crystalline structure. However, the dramatic peak decrease in both DSC and PXRD patterns revealed the partial amorphization and reduced crystallinity of CTS in nanocrystals (Figures 4, 5), which was mainly attributed to the reduction in crystal lattice energy caused by the incorporation of CTS in the hydrophobic domain of poloxamer 407 micelles and the complete surface coverage of particles by the stabilizer (Fontana et al., 2018). Additionally, amorphization/partial amorphization of drugs has been observed after lyophilization because solutes that cannot crystallize are converted to an amorphous state during the cooling step (Gol et al., 2018). Mannitol is a crystalline material with a characteristic endothermic peak at approximately 170°C. However, it disappeared in CTS products owing to the total amount of mannitol transformed into an amorphous form, which was also observed in other studies (Gol et al., 2018; Sharma and Mehta, 2019).

The main advantage of nanocrystals is the augmentation of the saturated solubility and subsequent dissolution rate of insoluble drugs. According to the Ostwald-Freundlich equation, the saturation solubility increases when the particle size is reduced below 1 µm (Buckton and Beezer, 1992). The tremendous rise in solubility at pH 1.2, pH 6.8, and water by CTS nanocrystals clearly confirmed the effect of particle size on drug solubility (Figure 6A). Meanwhile, nanocrystals can accelerate the dissolution rate as a consequence of the large interfacial area introduced by nanosized particles. The Noyes-Whitney equation precisely describes the relationship between the diffusion coefficient, surface area, dissolution volume, diffusion layer thickness and dissolution rate (Noyes and Whitney, 1897). The transformation of crystallinity to amorphous states, as we presented above in the PXRD and DSC spectra, contributed to rapid drug dissolution as well. Additionally, the surfactant poloxamer 407 not only acts as the crystal stabilizer, but also has low surface tension and small contact angle to promote the interaction of drug nanocrystals with the aqueous phase by wetting and dispersibility resulting in a quick dissolution profile (Meruva et al., 2020; Zhang et al., 2020). On the contrary, bulk CTS is hard to wetting because of its hydrophobicity. The release behavior of CTS nanocrystals in pH progressive media reflected the enhanced drug dissolution *in vitro* (Figure 6B), which endowed oral absorption in further *in vivo* studies.

Oral absorption is limited by the rate and extent of CTS dissolution. Therefore, improvement of CTS bioavailability (2.9-fold) was routinely observed once CTS nanocrystallization was achieved. Comparable CTS absorption was also reported to be enhanced by employing other delivery systems. Relative bioavailability of CTS loaded solid lipid nanoparticles was 186%–299% compared with CTS suspension depending on the lipid matrix and the amount of sodium dehydrocholate used in different formulations (Hu et al., 2010). Wheat germ agglutuinin-modified lipid-polymer hybrid nanoparticles could efficiently penetrate mucus layer and augment enterocyte uptake and transport *via* receptor-mediated endocytosis, resulting in 6.5-fold higher bioavailability than CTS suspension (Liu et al., 2020). Solid self-microemulsifying drug delivery system showed 499% relative bioavailability after oral administration in rats (Bi et al., 2016). The increased oral absorption was closely related to the substantial surfactants which were able to improve solubilization of CTS. Meanwhile, intestinal lipid bilayer membranes were also affected by these surfactants, resulting in the enhancement of drug permeability. The merit of CTS nanocrystals is simple composition, low surfactants comsuption, and easy preparation process. A high concentration gradient means fast diffusion of drug molecules across the intestinal membrane, which accounts for significantly higher C_max_ (Tab. 2) (Lv et al., 2022). In addition to the conventional mechanism, namely, enhanced solubility and dissolution rate, transepithelial transport of intact nanocrystals (Zhu et al., 2022), increased adhesiveness to the gastrointestnal tract wall with a large contact area of nanocrystals, along with enhanced permeability by poloxamer (Li et al., 2011), might also be involved in the enhancement of oral absorption. The T_max_ and MRT of raw CTS showed no obvious significant difference compared with those of the CTS nanocrystals.

## 5 Conclusion

CTS nanocrystals were successfully fabricated by an antisolvent precipitation-homogenization approach with a stabilizer of Poloxamer 407. Rational selection of the polymer concentration, homogenization pressure and number of cycles facilitated optimal control of the particle size. The freeze-dried product was stable for at least 6 months when stored at 4 °C. Optimized CTS nanocrystals had remarkable enhancement in solubility, dissolution rate and bioavailability over the raw material. Therefore, nanocrystals are a suitable delivery approach for CTS oral administration.

## Data Availability

The original contributions presented in the study are included in the article/supplementary material, further inquiries can be directed to the corresponding authors.
